# Translating the human microbiome: a path to improving health

**DOI:** 10.1186/s13073-021-00896-w

**Published:** 2021-05-05

**Authors:** Ramnik J. Xavier

**Affiliations:** 1 Center for Computational and Integrative Biology, Massachusetts General Hospital and Harvard Medical School, Cambridge Street, Boston, MA 02114 USA; 2 Center for Microbiome Informatics and Therapeutics, Massachusetts Institute of Technology, Carleton Street, Cambridge, MA 02139 USA

Translating the human microbiome in health and disease requires detailed information about each microbial member—from individual annotated genomes to community functional dynamics to relationships with the host. Toward this goal, phylogenetic descriptions of the microbiome are evolving into mechanistic analyses. Recent years have seen a dramatic rise in metagenomic and metabolomic studies using microbial-driven pathologies as models to understand host–microbiome interactions. These studies are charting community-wide ecological maps that provide insight into microbial determinants of health and disease, layering in impacts of the microbiome at different life stages. Although much is known about early microbiome development, trajectories during aging and later in life require further characterization.

The microbiome impacts every organ system and aspect of physiology, often through as-yet-unknown mechanisms. Moreover, the influence of the microbiome extends beyond the local environment: neuroactive molecules produced by gut commensals reach the brain to mediate mood, depression, and mental illness [1]. Understanding the principles that govern a healthy microbiome establishes a framework for interrogating perturbations associated with disease or diet and for developing interventional strategies. Unlike the genome, the microbiome is a dynamic reflection of health status that can be modified and, therefore, represents a largely untapped reservoir of opportunity to understand and exploit mechanisms that influence human physiology.

This special issue of *Genome Medicine* highlights the advances made in understanding strain diversity, microbial biochemistry, disease risk, and clinical outcomes. In this Editorial, I highlight key areas and recent progress that has been made in the field toward translating the microbiome and defining the many pathways that modulate host biology.

## Comprehensive maps from a single microbe

Metagenomic sequences provide a framework to generate testable mechanistic hypotheses; however, functional validation is limited by the availability of culturable isolates. Strain collections are being assembled, but those aiming to capture the breadth of diversity within the microbiome underrepresent strain-specific functional diversity. This obstacle is being circumvented in part by computational advancements, such as comparative genomics platforms that can analyze a single bacterial class, map genetic diversity to phenotypes, and identify potential competitive fitness genes through the quantification of highly related strains [2]. This approach highlights the value of comparative genomics in mechanistic analyses of unculturable microbes that impact human health.

## Microbiome and T cells

Recently, we have learnt that subsets of microbiome-reactive T cells are characteristic of health and various diseases, but our knowledge of microbial antigens is incomplete [3]. Computational tools are accelerating progress in antigen discovery, immunodominance, and characterizations of T cell receptors with functional phenotypes. Predicting antigen binding to MHC molecules, for instance, is a powerful approach to interrogate features of immunogenicity and specificity of T cell responses. The BOTA (bacteria originated T cell antigen) predictor uses genomic sequences to generate candidate MHCII-restricted epitopes based on accessibility criteria. When coupled with high-throughput validation for recognition by T cell receptors, it provides a platform to identify immunodominant epitopes. Detailed molecular characterizations of responses induced by bacterial isolates in T cell subsets harvested from healthy and disease tissues will aid future functional analyses.

Adaptive immunity is additionally affected by microbial metabolites, and recent work revisited the effects of bacterially modified bile acids on T cell biology. Lithocholic acid (LCA) derivatives were found to regulate the differentiation of naive T cells into Treg and Th17 cells, the balance of which plays a critical role in inflammatory bowel diseases (IBD) [4]. A network of microbial bile acid metabolites was shown to maintain a specific Treg population in the colon and ameliorates gut inflammation [5]. The secondary bile acid 3β-hydroxydeoxycholic acid (isoDCA) promoted Treg generation by reducing the immunostimulatory properties of dendritic cells [6]. On a global scale, the impact of the microbiome on host chemistry was revealed by comparing metabolomes from germ-free and specific-pathogen-free mice and uncovered previously unknown bile acid conjugates that are enriched in IBD and cystic fibrosis patients [7]. Toward unraveling microbial pathways responsible for bile acid metabolism, a set of six enzymes was found to be required to derive DCA and conferred the ability to produce DCA and LCA when expressed in a commensal that cannot synthesize these molecules naturally [8]. Thus, the potential for modulating metabolite levels in the host increases as knowledge of microbial biosynthetic pathways expands.

## Microbiome and diet

Dietary fibers modulate the composition and function of the microbiome, shaping host–microbiome interactions. Fiber-rich diets maintain a healthy, diverse microbiome and lead to beneficial metabolites such as short-chain fatty acids (SCFAs), which promote mucus and antimicrobial peptides production as well as maintain intestinal barrier integrity and robust immunity. Fiber-poor diets reduce diversity and alter microbial functions, impairing host physiology and increasing susceptibility to infection and chronic inflammation. Interventional increases in the dietary glycans, fructooligosaccharides and polydextrose, have been shown to cause quantifiable and consistent microbiome responses [9]. Importantly, decreases in the bacterially fermented metabolites of glycans, SCFAs, have been associated with IgE-mediated food allergy, providing an example of how dietary-driven microbiota changes can influence human health and disease [10].

Specific microbial commensals have been linked to beneficial health effects that are dependent on diet. An example is *Prevotella copri*, which has been correlated with improved glucose and insulin tolerance when subjects consume a high fiber diet [11, 12]. Two foundational studies have resolved genomic and functional variation in *Prevotella copri* and presented metagenomic analyses defining four carbohydrate-metabolizing clades highly prevalent in non-industrialized populations, which typically have high-fiber diets. Functional analyses confirmed that isolates from each clade utilize distinct sets of plant-derived polysaccharides. Thus, there is increasing interest in restoration of the healthy state potentially through dietary interventions or by utilizing microbial carbohydrate-active enzymes, CAZymes, to replace metabolic activity lost through diet-dependent microbiome changes.

## Microbiome and disease pathophysiology

Discoveries in microbiome-associated diseases point toward mechanisms underlying pathophysiology. Addressing concurrent increases in autoimmune and allergic disease rates, the hygiene hypothesis posits that early childhood exposure to certain microbes contributes to immune development and protects against these diseases. To test this, a longitudinal study followed children genetically predisposed to autoimmunity from birth to age three in neighboring countries [13]. Infants born in Finland and Estonia, where early-onset autoimmunity is common, were exposed to lipopolysaccharide (LPS) primarily from *Bacteroides* highly abundant in their microbiomes. Infants born in Russian Karelia, where autoimmunity is less prevalent, were exposed to a structurally distinct LPS from *Escherichia coli*. The study demonstrated that *Bacteroides* LPS inhibits innate immune activation by *E. coli* LPS, providing evidence that early life colonization by immune-silencing microbiota may hinder immune education and enhance susceptibility to autoimmune disease.

Gain- and loss-of-function mutations in C9orf72 contribute to neurodegenerative diseases including amyotrophic lateral sclerosis (ALS). Microbiomes of ALS patients differ from unaffected individuals, and recent evidence suggests that interactions between C9orf72 and the microbiome influence inflammatory responses within the nervous system. The latter stemmed from the observation that, despite identical genetic backgrounds, C9orf72-deficient mice reared in separate facilities harbored distinct microbiota and displayed marked differences in disease severity. A reduction in immune-stimulating bacteria protected C9orf72-deficient mice from neuroinflammation and early mortality, even after initial onset [14]. In contrast, disease was exacerbated in a Sod1-G93A mouse model of ALS lacking a microbiome or treated with antibiotics [15]. These findings underscore the importance of interactions between genetics and the microbiome in multifactorial diseases.

The microbiome influences cancer onset, progression, and response to therapy. While effects can be exerted indirectly through release of bioactive molecules into the circulation, it is also possible for the tumor microenvironment to support its own microbiome. Bacteria have long been detected in tumors, but the small biomass of the microenvironment made it challenging to exclude the possibility of contamination. A recent analysis surveyed bacteria in primary tumors and adjacent normal tissue across seven cancer types from body sites both exposed to and protected from microbes [16]. Each tumor type displayed a unique microbial profile, with breast tumors harboring the richest and most diverse microbiome. Microbes and their predicted metabolic functions associated with clinical features such as smoking status and response to therapy. In another example, using integrated multi-omics in hepatocellular carcinoma patients identified changes in the tumor immune microenvironment caused by the gut microbiota via serum bile acids, suggesting that gut microbes may be used as biomarkers of clinical features and outcomes [17]. Although determining mechanistic links remains a challenge, these studies represent important first steps.

## The missing microbiome and global conservancy

Current efforts are building extensive biobanks of human gut isolates with corresponding multi-omic data, not only to advance mechanistic research but to preserve and expand our understanding of microbiome biodiversity across communities worldwide. Research to date largely centers on industrialized populations, providing little insight into the microbiomes of non-industrialized populations that are often more diverse. Moreover, therapies based on or targeted for industrialized microbiomes might be ineffectual or even detrimental for other populations. Longitudinal microbiome analyses of the Hadza, an indigenous hunter-gatherer society, illuminated important roles played by gut microbes at risk of extinction from industrialization [18]. The Hadza diet follows distinct seasons that are reflected in microbiome composition and functional capacity, particularly in CAZymes. Taxa fluctuating most with season differentiated the Hadza from industrialized populations, suggesting that the presence of dynamic microbes decreased as a result of modern lifestyles. Isolate collections comprising strains from industrialized and non-industrialized populations are valuable not only to determine the impact of lifestyle on microbiome composition and diversity but also to investigate adaptation within bacterial genomes. Higher rates of horizontal gene transfer were found in the microbiomes of industrialized populations, indicating that microbes acquire new functionalities suited to the host lifestyle [19].

## Future directions

Human cohort studies will continue to be integral for translating the microbiome in health and disease. Treatment-naive cohorts and longitudinal studies revealed important alterations in microbiome community structure and function during IBD [3] and serve as templates for other microbiome-associated diseases. An early analysis of treatment-naive pediatric Crohn’s disease (CD) patients defined a microbial axis strongly associated with disease status and amplified by antibiotic treatment. This axis established the first link between oral microbes and chronic intestinal inflammation. Data from this cohort also correlated early anti-TNF therapy with reduced risk of certain complications and led to the development of a risk-stratification model. Studies of treatment-naive pediatric ulcerative colitis (UC) patients linked compositional and temporal microbiome changes with disease course and response to therapy. The Integrative Human Microbiome Project catalogued longitudinal taxonomic, functional, and biochemical shifts in IBD, identifying metabolites exclusively in IBD patients and uncovering distinct microbial enrichments in CD and UC [20]. Microbial and clinical factors implicated by these and other human cohort studies will inform optimal treatment strategies and guide clinical translations of the microbiome.

Multi-omic data exposed extensive “microbial dark matter” within the microbiome. Much about these unculturable microbes and their metabolic capabilities remains unknown, but new computational and biochemical approaches are enabling the characterization of unannotated molecules. The next steps will be to resolve the human metabolome into host, microbial, or co- metabolites (those initially derived from the host and modified by microbes, or vice versa) and build chemical libraries that can be tested in functional assays. A complete annotation of the microbes, genes, proteins, and metabolites within the human microbiome will be essential to hypothesis-based functional studies that reveal biological mechanisms at work in health and disease.

While a promising route to the improvement of human health, microbiome-based therapeutics face several current challenges. One is the need to study immunomodulatory molecules and other bioactives derived from microbes at physiological concentrations. Then, deep mechanistic understandings need to be developed with particular emphasis on potential pleiotropic effects. For example, a single microbial metabolite may bind multiple host receptors, each with a distinct, often cell type-specific biological outcome. Stimulation thresholds determined by signal strength, duration, or both might also dictate host responses. Synergistic or antagonistic effects that are not observed in reductionist models could appear in more complex contexts and emphasize the critical importance of conducting functional studies in simple systems, such as mono- and co-cultures, as well as sophisticated models that more closely resemble tissues. Lastly, pharmacokinetic processes of administration, absorption, distribution, metabolism, and excretion need to be considered when evaluating metabolites or microbes themselves as therapeutics. The future of microbiome-based precision medicine will rely on extensive hypothesis-based functional work, as well as full consideration of the complexities between microbes and their hosts, in order to improve human health.

## Data Availability

Not applicable.
